# Medical Rhymes

**Published:** 1884-12

**Authors:** 


MEDICAL RHYMES, a collection of Rhymes selected and compiled from a variety of sources. By Hugo Erichsen, M. D., Professor of Neurology in the Quincy Schools of Medicine, Medical Department of Chaddock College, Licentiate of Royal College of Physicians and Surgeons of Kingston, Canada, member of the Detroit Medical and Library Association; Corresponding member of the Natural History of Wisconsin, etc., with an introduction by Professor Willis P. King, M. D., Sedalia Mo., ex-President of the Missouri State Medical Society, etc. Illustrated. J. H. Chambers & Co., St. Louis, Chicago and Atlanta. 1884.

This is a handsomely bound book of 220 pages, embellished with 19 well executed engravings. It is a novelty in book-making. The author in his preface says, "The purpose of my book is to amuse the busy doctor in his leisure hours." And right well has he accomplished his purpose. We commend the work to our readers as one worthy of perusal.

